# Dexmedetomidine-Based Versus Opioid-Containing Periarticular Infiltration in High Tibial Osteotomy: A Retrospective Comparative Cohort Study

**DOI:** 10.3390/healthcare14162580

**Published:** 2026-08-17

**Authors:** Hasan Emirhan Usta, Ruhat Ünlü, Yıldıray Tekçe, Giray Tekçe

**Affiliations:** 1 Department of Orthopedics and Traumatology, Gebze Fatih State Hospital, Kocaeli 41400, Turkey; mdemirhanusta@gmail.com (H.E.U.); yildiraytekce@gmail.com (Y.T.); yarig2906@gmail.com (G.T.); 2 Department of Orthopedics and Traumatology, Istanbul Atlas University, Istanbul 34403, Turkey

**Keywords:** high tibial osteotomy, dexmedetomidine, periarticular infiltration analgesia, modified Ranawat cocktail, opioid-sparing analgesia, morphine milligram equivalents, postoperative pain, patient-controlled analgesia

## Abstract

**Highlights:**

**What are the main findings?**

- Dexmedetomidine-based opioid-free periarticular infiltration was associated with lower VAS scores during the 12–24 h postoperative period after medial opening-wedge high tibial osteotomy.
- The dexmedetomidine-based protocol was associated with reduced 24 h systemic opioid consumption and fewer PCA demands compared with the modified opioid-containing Ranawat cocktail.

**What are the implications of the main findings?**

- Dexmedetomidine-based periarticular infiltration may represent a promising opioid-sparing component of multimodal analgesia after HTO.
- Because of the retrospective non-randomized design and limited availability of some confounding variables, these findings should be interpreted as associative and hypothesis-generating.

**Abstract:**

**Background/Objectives:** High tibial osteotomy (HTO) is a joint-preserving procedure for selected patients with medial compartment knee osteoarthritis and varus malalignment. Postoperative pain after HTO may impair early mobilization and increase systemic opioid requirements. Periarticular infiltration analgesia is commonly used as part of multimodal analgesia; however, comparative evidence regarding opioid-containing and opioid-free infiltration protocols in HTO remains limited. This study compared postoperative pain, systemic opioid consumption, PCA demand, rescue analgesic requirement, and analgesia-related adverse events between a modified opioid-containing Ranawat cocktail and an opioid-free dexmedetomidine-based periarticular infiltration protocol. **Methods:** This single-center retrospective comparative cohort study included 80 patients who underwent medial opening-wedge HTO between March 2021 and March 2025. Patients were categorized according to the documented periarticular infiltration protocol: modified Ranawat cocktail (n = 40) or dexmedetomidine-based cocktail (n = 40). Postoperative VAS scores were recorded at 6, 12, 18, and 24 h and on postoperative days 7 and 14. Secondary outcomes included 24 h systemic opioid consumption expressed as morphine milligram equivalents, PCA demand count, rescue analgesic use, and analgesia-related adverse events. Longitudinal postoperative VAS trajectories were analyzed using unadjusted and adjusted linear mixed-effects models. The adjusted model included the available covariates of age, body mass index, preoperative VAS score, operative time, and tourniquet time. **Results:** Baseline demographic and perioperative variables were comparable between groups. The dexmedetomidine group demonstrated lower adjusted postoperative VAS scores during the early postoperative period, with statistically significant between-group differences at 12, 18, and 24 h, whereas pain trajectories converged by postoperative day 14. Twenty-four-hour systemic opioid consumption was lower in the dexmedetomidine group than in the modified Ranawat group (9.78 ± 1.99 vs. 13.92 ± 1.79 MME, *p* < 0.001). PCA demand was also lower in the dexmedetomidine group (6.30 ± 3.16 vs. 9.63 ± 2.92, *p* < 0.001). No significant between-group differences were observed in rescue NSAID use, rescue tramadol requirement, nausea/vomiting, hypotension, bradycardia, allergic reactions, or other recorded adverse events. **Conclusions:** In this retrospective cohort, dexmedetomidine-based opioid-free periarticular infiltration was associated with lower adjusted early postoperative VAS scores, reduced 24 h systemic opioid consumption, and fewer PCA demands compared with a modified opioid-containing Ranawat cocktail after medial opening-wedge HTO. However, because treatment allocation was not randomized and several potentially relevant confounders were not available for adjustment, these findings should be interpreted as associative and hypothesis-generating. Prospective adequately powered randomized studies are needed to confirm these findings and evaluate their effects on functional recovery and osteotomy healing.

## 1. Introduction

High tibial osteotomy (HTO) is a well-established joint-preserving procedure for selected patients with medial compartment knee osteoarthritis associated with varus malalignment. By shifting the mechanical axis laterally and reducing excessive medial compartment loading, HTO aims to relieve pain, improve function, and delay or avoid knee arthroplasty in appropriately selected active patients [1,2]. Although correction accuracy, fixation stability, and postoperative alignment remain key determinants of clinical success, the early postoperative period is also clinically important because pain control may influence mobilization, rehabilitation participation, opioid requirement, and patient satisfaction. Postoperative pain after HTO can be considerable because the procedure involves osteotomy creation, periosteal manipulation, soft-tissue dissection, implant fixation, and postoperative mechanical changes around the proximal tibia. Inadequate analgesia during the first postoperative hours and days may delay mobilization and increase reliance on systemic opioids. Contemporary postoperative pain management guidelines emphasize multimodal, opioid-sparing analgesic strategies tailored to the surgical procedure rather than relying on universal analgesic protocols [3]. This approach is particularly relevant in joint-preserving knee surgery, where early functional recovery is an important component of treatment success. Local or periarticular infiltration analgesia has become an important component of multimodal pain management in knee surgery. In total knee arthroplasty, local infiltration analgesia has been widely studied and has been associated with reductions in early postoperative pain and opioid consumption, although the optimal combination of agents remains variable across institutions [4,5]. However, evidence from arthroplasty should not be extrapolated uncritically to HTO, because the two procedures differ in surgical trauma, pain generators, rehabilitation pathways, and biological priorities. In particular, HTO involves an osteotomy site where preservation of bone healing is a fundamental therapeutic objective, which may influence the selection of periarticular analgesic adjuvants. HTO-specific evidence regarding periarticular infiltration analgesia remains limited. Jung et al. reported that periarticular multimodal drug injection reduced early postoperative pain after medial opening-wedge HTO, supporting the potential value of local infiltration strategies in this specific setting [6]. Nevertheless, the optimal composition of periarticular infiltration protocols after HTO has not been clearly established. Modified Ranawat-type cocktails, originally developed and widely used in arthroplasty practice, typically include a local anesthetic, a nonsteroidal anti-inflammatory drug, epinephrine, and an opioid component such as morphine [5]. In the context of increasing emphasis on opioid stewardship, the continued use of opioid-containing local infiltration protocols warrants further evaluation, particularly in procedures where opioid-free alternatives may provide adequate analgesia while reducing systemic opioid requirements. Dexmedetomidine, a highly selective α_2_-adrenergic agonist, has attracted increasing attention as an analgesic adjunct in regional anesthesia and local infiltration protocols. Its potential analgesic effects include modulation of nociceptive transmission, prolongation of local anesthetic action, and reduction in systemic opioid requirements. Recent studies in total knee arthroplasty suggest that adding dexmedetomidine to periarticular infiltration analgesia may prolong early analgesic effects and improve recovery-related outcomes without substantially increasing adverse events [7,8]. However, these findings are largely based on arthroplasty populations, and evidence regarding dexmedetomidine-based opioid-free periarticular infiltration in patients undergoing HTO remains limited. Taken together, the available literature reveals three important gaps. First, HTO-specific comparative evidence regarding periarticular infiltration analgesia remains limited compared with the arthroplasty literature. Second, the clinical role of dexmedetomidine as a periarticular infiltration adjunct after HTO has not been sufficiently evaluated. Third, comparative data between opioid-containing modified Ranawat protocols and opioid-free dexmedetomidine-based protocols in HTO are lacking. Therefore, the primary objective of this retrospective comparative cohort study was to compare postoperative pain trajectories assessed by serial VAS measurements between patients receiving a modified opioid-containing Ranawat cocktail and those receiving an opioid-free dexmedetomidine-based periarticular infiltration protocol after medial opening-wedge HTO. Secondary outcomes included 24 h systemic opioid consumption, PCA demand count, rescue analgesic requirement, and analgesia-related adverse events. We hypothesized that the dexmedetomidine-based opioid-free protocol would be associated with lower early postoperative pain trajectories and reduced systemic opioid requirements compared with the modified opioid-containing Ranawat protocol.

## 2. Materials and Methods

### 2.1. Study Design, Setting, and Ethical Approval

This single-center retrospective comparative cohort study was conducted at the Department of Orthopaedics and Traumatology, Gebze Fatih State Hospital, Kocaeli, Türkiye. The study included consecutive adult patients who underwent medial opening-wedge high tibial osteotomy (HTO) between March 2021 and March 2025. The study protocol was approved by the Istanbul Atlas University Scientific Research Ethics Committee. The protocol was reviewed at the committee meeting held on 24 November 2025, and official approval was issued on 27 November 2025 (Decision No: 10/53; Approval Document No: E-22686390-050.99-84338). Because this was a retrospective analysis based exclusively on anonymized existing clinical records, the requirement for individual written informed consent was waived by the ethics committee. No additional procedures, interventions, or direct patient contact were performed for the purpose of this study. The study was conducted in accordance with the principles of the Declaration of Helsinki and was reported in accordance with the Reporting of studies Conducted using Observational Routinely collected health Data (RECORD) statement [9]. A completed RECORD checklist is provided as Supplementary Table S1.

### 2.2. Study Population and Eligibility Criteria

**Figure 1:**
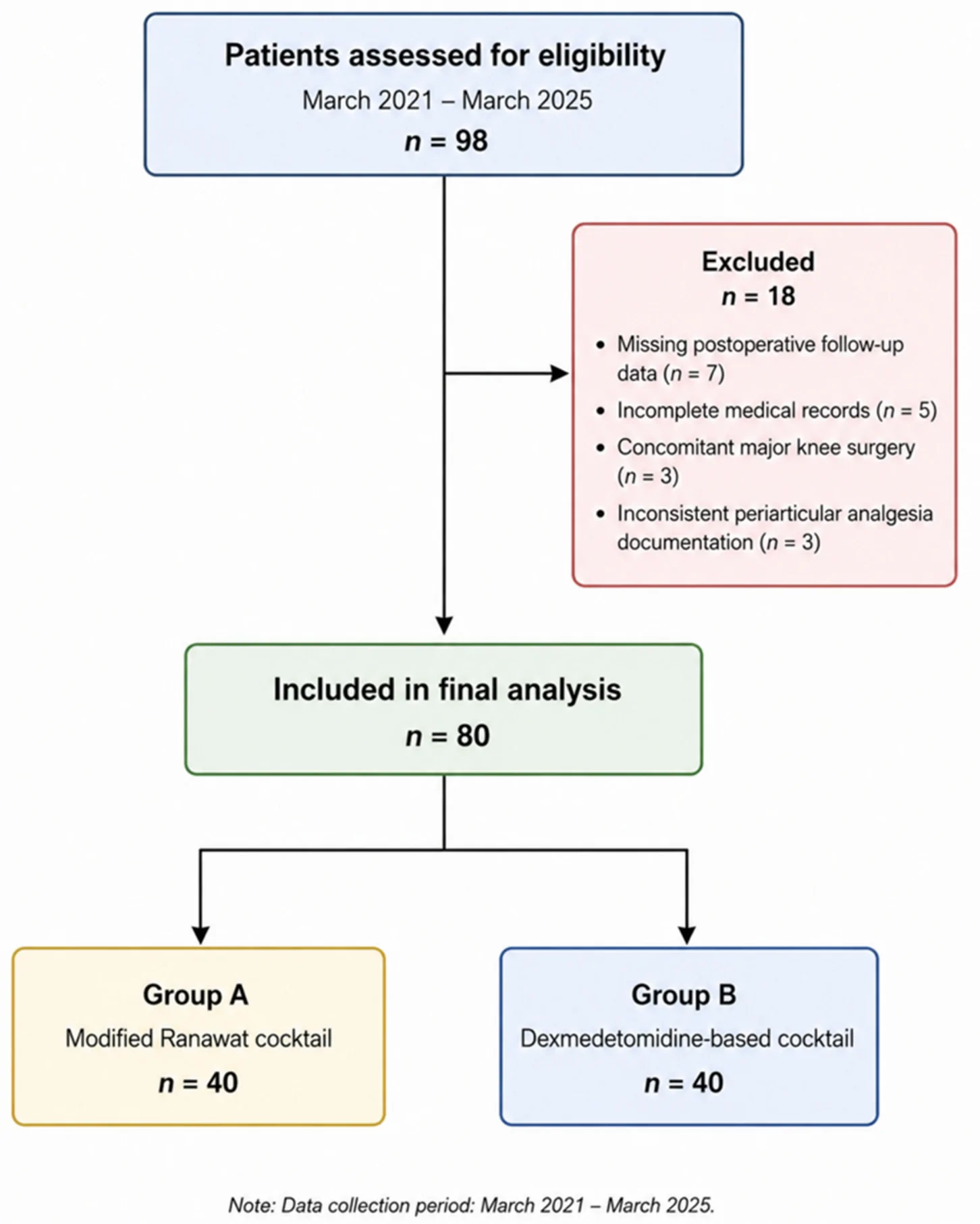
Flow diagram of patient selection and study inclusion.

The study population consisted of consecutive adult patients who underwent medial opening-wedge HTO during the predefined study period. Eligible patients were identified through institutional electronic medical records, operative reports, anesthesia records, and postoperative nursing observation charts. A total of 98 patients were screened for eligibility. After application of the predefined inclusion and exclusion criteria, 80 patients were included in the final analysis. Patients were categorized according to the documented periarticular infiltration analgesia protocol into the modified Ranawat cocktail group (n = 40) or the dexmedetomidine-based cocktail group (n = 40). The patient selection process is shown in Figure 1.

Because of the retrospective observational design, all consecutive patients meeting the eligibility criteria during the study period were included without prospective recruitment or sampling. Accordingly, the final sample size was determined by the number of eligible patients available within the predefined study period rather than by a prospective enrollment target.

The inclusion criteria were as follows:

- Age ≥ 18 years;
- Primary medial opening-wedge HTO performed for symptomatic medial compartment knee osteoarthritis with varus malalignment;
- Clearly documented intraoperative periarticular infiltration analgesia protocol;
- Complete postoperative pain assessment records at 6, 12, 18, and 24 h and on postoperative days 7 and 14;
- Complete perioperative and postoperative analgesic documentation, including opioid consumption, PCA use, and rescue analgesic administration.

Patients were excluded if they had:

- Revision HTO;
- Concomitant major knee surgery likely to affect postoperative pain, such as ligament reconstruction, revision osteotomy, or arthroplasty;
- Incomplete or unreliable perioperative records;
- Undocumented, unclear, or inconsistent periarticular infiltration analgesia documentation;
- Missing postoperative pain assessments;
- Incomplete follow-up data required for the predefined outcome measures.

### 2.3. Surgical Technique and Periarticular Infiltration Protocols

All procedures were performed by the same senior orthopedic surgeon using a standardized medial opening-wedge HTO technique. After diagnostic arthroscopy, a biplanar medial opening-wedge osteotomy was created at the proximal tibia under fluoroscopic guidance using a Genoray C-arm system (GENORAY Co., Ltd., Seongnam-si, Republic of Korea) while preserving the lateral cortical hinge. The osteotomy gap was gradually opened until the planned mechanical axis correction was achieved and was stabilized using the same medial high tibial locking plate system (Çekiçoğlu Makina Medikal, Izmir, Turkey) in all patients. The same surgical approach, osteotomy technique, fixation principles, implant system, intraoperative correction strategy, perioperative surgical workflow, and postoperative rehabilitation pathway were used throughout the study period. Bone grafting was performed according to the intraoperative requirements of the osteotomy gap. Because detailed radiographic correction parameters, exact opening gap measurements, and bone grafting details were not consistently available in the retrospective records, these variables could not be included in the adjusted analyses and were considered during interpretation of the findings. Following fixation and immediately before wound closure, periarticular infiltration analgesia was administered using a standardized infiltration technique. The solution was infiltrated into the periosteum, joint capsule, surrounding soft tissues, and tissues adjacent to the osteotomy site. The anatomical injection sites and infiltration technique were identical between groups. Patients received one of two institutionally accepted periarticular infiltration protocols: Group A: Modified Ranawat cocktail: Patients in Group A received a steroid-free modified Ranawat cocktail consisting of 0.2% ropivacaine, ketorolac 30 mg, morphine sulfate 2–5 mg, and epinephrine 1:200,000. The total injection volume was standardized to 60 mL using isotonic saline dilution. Group B: Dexmedetomidine-based cocktail: Patients in Group B received an opioid-free dexmedetomidine-based cocktail consisting of 0.2% ropivacaine, ketorolac 30 mg, dexmedetomidine 50–100 μg, and epinephrine 1:200,000. The total injection volume was standardized to 60 mL using isotonic saline dilution. Variations in morphine and dexmedetomidine doses within the predefined institutional dose ranges reflected routine clinical practice. Because exact administered dose distributions were not consistently available for all patients, dose–response analyses could not be performed. This was considered a potential source of within-group heterogeneity.

### 2.4. Group Allocation and Bias Considerations

**Figure 2:**
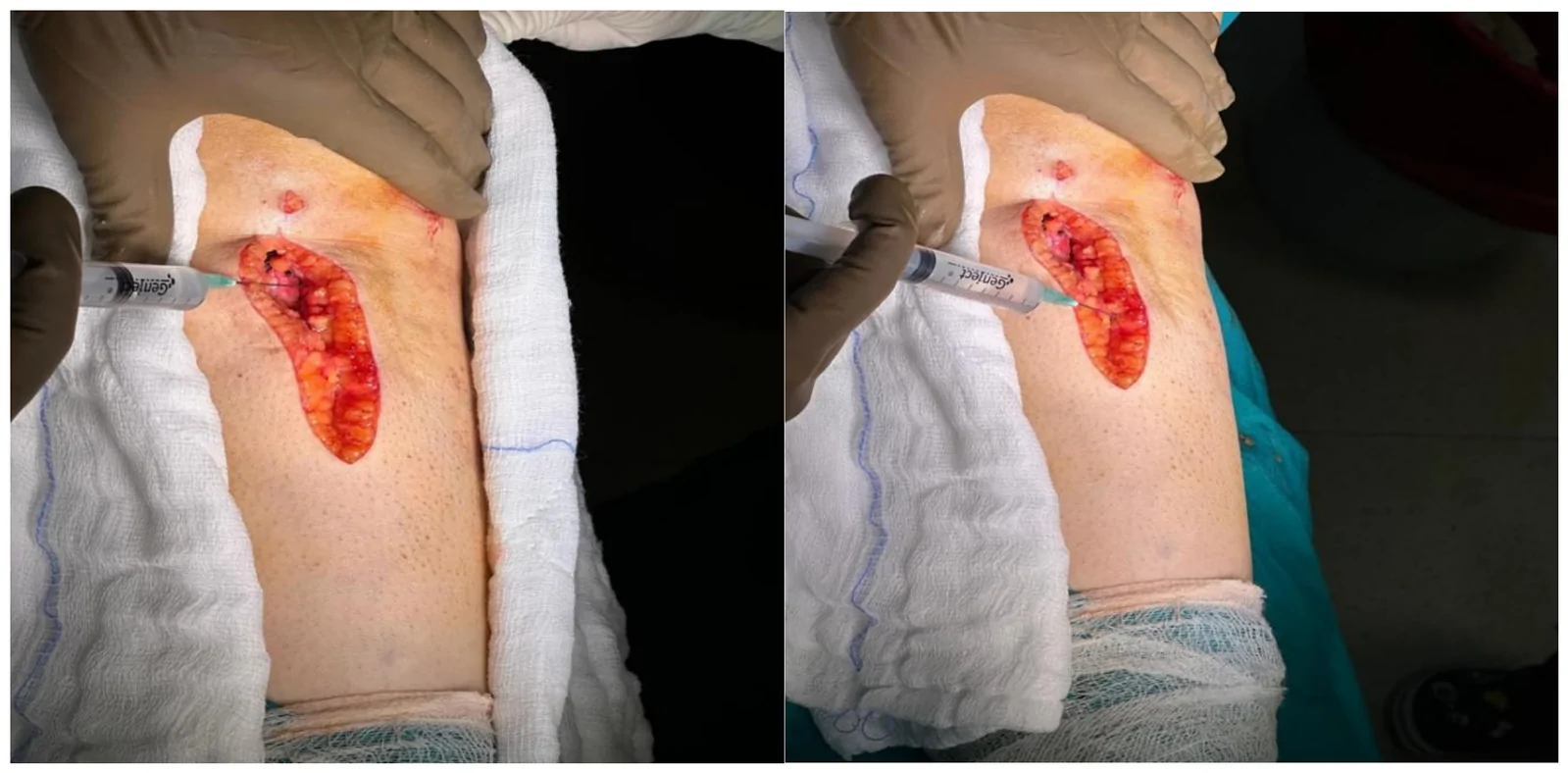
Intraoperative periarticular infiltration analgesia technique during high tibial osteotomy. The analgesic solution was infiltrated into the periosteum, capsule, and surrounding soft tissues around the osteotomy site before wound closure.

Patients were retrospectively assigned to the study groups according to the periarticular infiltration analgesia protocol documented in the operative and anesthesia records. Because of the retrospective observational design, treatment allocation was not prospectively planned, randomized, or concealed. Both periarticular infiltration protocols were used concurrently throughout the study period as part of routine clinical practice rather than being introduced sequentially (Figure 2). Therefore, group allocation was not associated with a chronological change in institutional practice, reducing the likelihood of historical or time-period bias. Selection of the periarticular infiltration protocol was surgeon-directed and reflected routine clinical practice within two institutionally accepted analgesic protocols. No predefined allocation algorithm based on patient age, sex, body mass index, comorbidity status, disease severity, or baseline pain score was used. The analgesic protocol was selected during routine clinical care before postoperative outcomes were known and was not assigned for research purposes. To reduce information bias, only patients with clearly documented intraoperative infiltration protocols and complete perioperative analgesia records were included. Baseline demographic and perioperative characteristics were compared between groups to evaluate comparability. Despite these measures, the retrospective non-randomized design remains an inherent limitation. Residual selection bias, confounding by indication, and unmeasured confounding cannot be completely excluded. Therefore, observed between-group differences should be interpreted as associations rather than definitive evidence of causal superiority.

### 2.5. Perioperative Anesthesia, PCA Protocol, and Postoperative Rehabilitation

All patients underwent standardized spinal anesthesia using 12.5 mg hyperbaric bupivacaine according to the institutional anesthesia protocol. General anesthesia and peripheral nerve block techniques were not routinely used in the study population. Supplemental intravenous sedation was administered when clinically indicated during surgery; however, detailed sedation exposure and dose data were not consistently available in the retrospective records and therefore could not be included in the adjusted analyses. Postoperative analgesia was standardized for all patients. Intravenous patient-controlled analgesia (PCA) with tramadol was initiated immediately after surgery in all patients. PCA settings were identical between groups and consisted of a 20 mg bolus dose, a 20 min lockout interval, no background infusion, and a maximum cumulative dose of 400 mg during the first 24 postoperative hours. PCA demand count represented all patient-triggered button presses recorded by the PCA device, including unsuccessful demands made during the lockout interval. Additional rescue analgesics, including supplemental tramadol and nonsteroidal anti-inflammatory drugs (NSAIDs), were administered when clinically indicated according to the institutional postoperative pain management protocol. Rescue analgesic administration was determined by the treating clinical team according to routine postoperative clinical assessment and documented in the medication administration records. Because rescue analgesic administration was based on routine clinical judgment rather than a strictly protocolized VAS threshold, this was considered a limitation and a potential source of provider-related variability. Postoperative rehabilitation was identical for both groups. All patients followed the same standardized rehabilitation pathway, including early mobilization, range-of-motion exercises, progressive weight-bearing, physiotherapy recommendations, inpatient nursing care, discharge criteria, and scheduled outpatient follow-up. Accordingly, perioperative management other than the periarticular infiltration protocol was standardized as much as possible throughout the study period.

### 2.6. Assessment of Postoperative Opioid Consumption

Postoperative systemic opioid consumption was obtained from routine postoperative medication administration records and expressed as morphine milligram equivalents (MME). Twenty-four-hour systemic opioid consumption represented the cumulative systemic opioid requirement during the first 24 postoperative hours. Intravenous tramadol administered through the PCA device constituted the primary postoperative opioid. When rescue tramadol was administered, the doses actually administered were incorporated into cumulative systemic opioid consumption. Tramadol consumption was converted to morphine milligram equivalents (MME) using a conversion factor of 100 mg tramadol = 10 mg MME, based on established opioid equivalence conversion tables [10]. Non-opioid rescue analgesics, including NSAIDs, were not included in the MME calculation and were analyzed separately as rescue analgesic use. Morphine administered locally as part of the modified Ranawat periarticular infiltration cocktail was excluded from systemic MME calculations because the outcome was intended to quantify postoperative systemic opioid requirements rather than the opioid administered as part of the local infiltration intervention.

### 2.7. Outcome Measures and Data Collection

The primary outcome was postoperative pain intensity assessed using the Visual Analog Scale (VAS). Pain scores were routinely recorded at 6, 12, 18, and 24 postoperative hours and during outpatient follow-up on postoperative days 7 and 14. Early postoperative VAS scores at 6, 12, 18, and 24 h were obtained from inpatient nursing observation charts. Postoperative day 7 and day 14 VAS scores were obtained from routine outpatient follow-up records in the electronic medical record system (BilMedical^®^ Hospital Information Management System, v30.125, Bilmed, Turkey). VAS data were available for all 80 patients at all predefined postoperative time points. Secondary outcomes included 24 h cumulative systemic opioid consumption expressed as MME, PCA demand count, rescue NSAID use, rescue tramadol requirement, analgesia-related adverse events, and length of hospital stay, which was evaluated as an exploratory outcome because it was not a prespecified study outcome. Recorded adverse events included nausea/vomiting, hypotension, bradycardia, allergic reactions, local wound-related complications, and other clinically documented analgesia-related events. Clinical data were retrospectively extracted from the institutional electronic medical record system, anesthesia records, operative reports, inpatient nursing observation charts, medication administration records, and outpatient follow-up documentation. Data extraction was performed independently by two investigators using a standardized data collection form. Patient identifiers were removed before analysis and replaced with anonymized study codes. Any discrepancies identified during data extraction were resolved by joint review of the original medical records. Several potentially relevant confounding variables were not consistently or reliably documented in the retrospective records and therefore could not be included in the adjusted analyses because they were unavailable for all patients. These included ASA physical status, detailed comorbidity profiles, chronic opioid use, preoperative analgesic consumption, psychiatric comorbidity, detailed sedation exposure, radiographic correction parameters, exact osteotomy opening gap, bone grafting details, and minor concomitant arthroscopic procedures.

### 2.8. Statistical Analysis

All statistical analyses were performed using IBM SPSS Statistics for Windows, version 26.0 (IBM Corp., Armonk, NY, USA). Continuous variables were evaluated for normality using visual inspection of histograms and Q–Q plots together with the Shapiro–Wilk test. Normally distributed continuous variables are presented as mean ± standard deviation (SD), whereas non-normally distributed variables are expressed as median and interquartile range (IQR) or median with minimum–maximum values, as appropriate. Categorical variables are presented as frequencies and percentages. Baseline demographic and perioperative characteristics were compared between groups using the independent-samples t-test or Mann–Whitney U test for continuous variables, as appropriate. Categorical variables were compared using the χ^2^ test or Fisher’s exact test when expected cell counts were low. Longitudinal postoperative VAS scores were analyzed using a linear mixed-effects model to account for repeated measurements within individuals. Treatment group, postoperative time, and the group × time interaction were included as fixed effects. Patient identification was included as a random intercept to account for within-subject correlation. Restricted maximum likelihood (REML) estimation was used for parameter estimation. An unstructured residual covariance structure was specified for the repeated-measures data to account for within-subject correlations across serial postoperative VAS measurements because it provided the best fit for the observed covariance pattern. All mixed-effects models converged successfully. Estimated marginal means with 95% confidence intervals were calculated from the adjusted linear mixed-effects model for each postoperative time point. The primary hypothesis focused on the group × time interaction, which evaluated whether postoperative pain trajectories differed between the two periarticular infiltration protocols over time. Pairwise comparisons between treatment groups at the six predefined postoperative time points (6, 12, 18, and 24 h and postoperative days 7 and 14) were performed using Bonferroni-adjusted significance levels. To account for available measured confounding, an adjusted linear mixed-effects model was additionally constructed including age, body mass index, preoperative VAS score, operative time, and tourniquet time as fixed covariates. Because of the retrospective design, adjustment was limited to covariates that were consistently available in the medical records. Other potentially relevant confounders, including ASA physical status, detailed comorbidities, preoperative opioid or analgesic use, sedation exposure, and radiographic correction parameters, were not consistently documented and therefore could not be included in the adjusted models. For secondary continuous outcomes, including total 24 h MME and PCA demand count, between-group differences were evaluated using appropriate parametric or non-parametric tests according to data distribution. When appropriate, adjusted analyses of covariance were performed using the same available covariates: age, body mass index, preoperative VAS score, operative time, and tourniquet time. Effect sizes were reported using Cohen’s d for continuous between-group comparisons where appropriate. A two-sided *p* value < 0.05 was considered statistically significant.

## 3. Results

### 3.1. Patient Flow

A total of 98 patients who underwent medial opening-wedge high tibial osteotomy during the predefined study period were screened for eligibility. Eighteen patients were excluded because of missing postoperative follow-up data (n = 7), incomplete medical records (n = 5), concomitant major knee surgery (n = 3), or inconsistent periarticular analgesia documentation (n = 3). The final analysis included 80 consecutive eligible patients, categorized according to the documented periarticular infiltration protocol into the modified Ranawat cocktail group (n = 40) and the dexmedetomidine-based cocktail group (n = 40) (Figure 1).

### 3.2. Baseline and Perioperative Characteristics

Baseline demographic and perioperative characteristics are summarized in Table 1. The two groups were comparable with respect to the available baseline demographic and perioperative characteristics, including age, sex distribution, body mass index, operated side, preoperative VAS score, operative time, and tourniquet time. No statistically significant between-group differences were observed in any of the available baseline or perioperative variables (Table 1).

### 3.3. Postoperative Pain Trajectories

**Figure 3:**
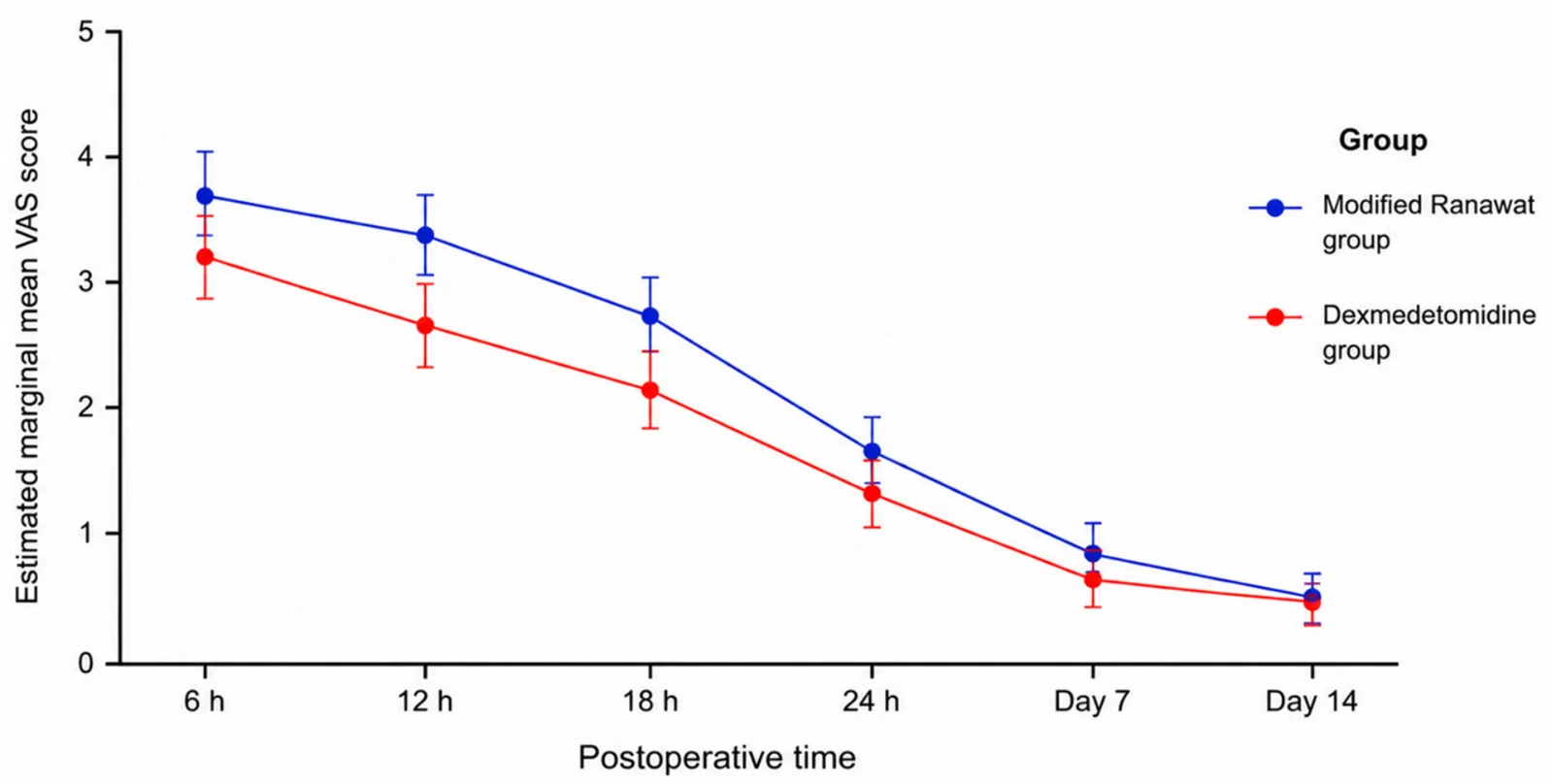
Estimated marginal mean postoperative VAS scores over time according to periarticular infiltration protocol.

VAS data were available for all 80 patients at all predefined postoperative time points, including 6, 12, 18, and 24 h, as well as postoperative days 7 and 14. Postoperative VAS scores decreased progressively over time in both treatment groups. Linear mixed-effects model analysis demonstrated a significant main effect of treatment group (F = 4.79, df = 1, *p* = 0.032), a significant main effect of postoperative time (F = 380.97, df = 5, *p* < 0.001), and a significant group × time interaction (F = 7.75, df = 5, *p* < 0.001), indicating that postoperative pain trajectories differed between the two periarticular infiltration protocols. After adjustment for the available covariates, including age, body mass index, preoperative VAS score, operative time, and tourniquet time, the group × time interaction remained statistically significant (F = 7.78, df = 5, *p* < 0.001), indicating that the observed difference persisted after adjustment for the measured variables available in the retrospective dataset. Estimated marginal means derived from the adjusted linear mixed-effects model demonstrated lower postoperative VAS scores in the dexmedetomidine group throughout the early postoperative period. Pairwise comparisons demonstrated statistically significant between-group differences at 12 h (mean difference, 0.96 VAS points; 95% CI, 0.48–1.44; Bonferroni-adjusted *p* < 0.001), 18 h (mean difference, 0.88 VAS points; 95% CI, 0.40–1.36; Bonferroni-adjusted *p* = 0.001), and 24 h (mean difference, 0.66 VAS points; 95% CI, 0.18–1.14; Bonferroni-adjusted *p* = 0.008). No statistically significant between-group differences were observed at 6 h, postoperative day 7, or postoperative day 14 after Bonferroni adjustment. These findings indicate that the greatest analgesic benefit occurred during the first postoperative 24 h, with progressive convergence of pain trajectories thereafter (Table 2; Figure 3).

Error bars represent 95% confidence intervals derived from the adjusted linear mixed-effects model. The dexmedetomidine-based periarticular infiltration group demonstrated significantly lower estimated postoperative VAS scores at 12, 18, and 24 postoperative hours, with progressive convergence of pain trajectories by postoperative day 14.

### 3.4. Opioid Consumption, PCA Demand, Rescue Analgesic Use, and Adverse Events

Postoperative analgesic outcomes, exploratory length of hospital stay, and analgesia-related adverse events are summarized in Table 3. Total 24 h systemic opioid consumption was significantly lower in the dexmedetomidine group than in the modified Ranawat group (9.78 ± 1.99 vs. 13.92 ± 1.79 MME; mean difference, 4.14 MME; 95% CI, 3.30–4.98; *p* < 0.001). Similarly, PCA demand count was significantly lower in the dexmedetomidine group (6.30 ± 3.16 vs. 9.63 ± 2.92; mean difference, 3.33; 95% CI, 1.98–4.68; *p* < 0.001). No statistically significant between-group differences were observed in rescue NSAID use, rescue tramadol requirement, nausea/vomiting, hypotension, bradycardia, allergic reactions, or other recorded adverse events. Length of hospital stay was statistically shorter in the dexmedetomidine group; however, the absolute difference was small (2.29 ± 0.30 vs. 2.46 ± 0.33 days; mean difference, 0.17 days; 95% CI, 0.03–0.31; *p* = 0.017), and this finding should be interpreted cautiously.

### 3.5. Adjusted Analyses for Secondary Outcomes

To evaluate whether the observed between-group differences in postoperative opioid consumption and PCA demand remained after adjustment for the available measured covariates, analyses of covariance (ANCOVA) were performed using age, body mass index, preoperative VAS score, operative time, and tourniquet time as covariates. Detailed adjusted ANCOVA results for total 24 h opioid consumption and PCA demand count are provided in Supplementary Table S2. After adjustment for available measured covariates, treatment group remained significantly associated with total 24 h MME (F = 98.33, *p* < 0.001, partial η^2^ = 0.574) and PCA demand count (F = 21.54, *p* < 0.001, partial η^2^ = 0.228). Among the included covariates, body mass index showed a modest association with total 24 h MME (F = 4.07, *p* = 0.047), whereas age, preoperative VAS score, operative time, and tourniquet time were not significantly associated with opioid consumption. For PCA demand count, none of the included covariates, including age, body mass index, preoperative VAS score, operative time, or tourniquet time, showed a statistically significant association. Because adjustment was limited to covariates consistently available in the retrospective records, these findings should be interpreted as adjusted associations based on measured available variables rather than comprehensive control of all clinically relevant confounders or definitive evidence of causality.

## 4. Discussion

The present retrospective comparative cohort study evaluated an opioid-containing modified Ranawat periarticular infiltration protocol and an opioid-free dexmedetomidine-based periarticular infiltration protocol in patients undergoing medial opening-wedge high tibial osteotomy. The main finding was that the dexmedetomidine-based protocol was associated with lower early postoperative VAS scores, particularly during the 12–24 h postoperative period, together with reduced 24 h systemic opioid consumption and fewer PCA demands. However, the absolute between-group differences in VAS scores were modest, and pain trajectories converged by postoperative day 14. Therefore, the analgesic advantage observed in this study should be interpreted primarily as an early postoperative opioid-sparing association rather than definitive evidence of broad clinical superiority.

Effective postoperative analgesia after HTO is clinically important because the procedure involves osteotomy creation, periosteal manipulation, soft-tissue dissection, implant fixation, and early rehabilitation demands. Contemporary postoperative pain management emphasizes procedure-specific multimodal analgesia and opioid minimization rather than reliance on systemic opioids alone [3]. In this context, periarticular infiltration analgesia may be particularly relevant for HTO because it directly targets local pain generators around the osteotomy site and surrounding soft tissues. Nevertheless, HTO-specific evidence remains limited compared with the extensive arthroplasty literature. Jung et al. reported that periarticular multimodal drug injection reduced postoperative pain after medial opening-wedge HTO, supporting the value of local infiltration strategies in this specific setting [6]. The present study extends this concept by comparing two different infiltration compositions rather than evaluating infiltration versus no infiltration.

The temporal pattern of postoperative pain observed in this study is clinically and pharmacologically plausible. At 6 h postoperatively, VAS scores were relatively similar between groups, which may be explained by the residual effects of spinal anesthesia, systemic postoperative analgesia, and the local anesthetic component common to both infiltration protocols. Between 12 and 24 h, the dexmedetomidine-based group showed lower estimated VAS scores, suggesting that the addition of dexmedetomidine may have prolonged or stabilized the early analgesic effect after the initial anesthetic phase. This pattern is consistent with the concept of a postoperative rebound phenomenon after the waning of local anesthetic effects, as described in the HTO periarticular injection literature [6]. By postoperative day 7 and day 14, pain scores were low in both groups and no longer meaningfully separated, indicating that the potential benefit of dexmedetomidine-based infiltration was mainly limited to the early postoperative period.

Although the VAS differences favored the dexmedetomidine-based protocol during the early postoperative period, their absolute magnitude was modest. This point is important because statistically significant differences in pain scores do not always translate into clinically meaningful differences. Therefore, the clinical interpretation of the VAS results should be cautious. In contrast, the reduction in 24 h systemic opioid consumption and PCA demand provides a more clinically interpretable signal. Lower opioid exposure is relevant in orthopedic postoperative care because opioids may contribute to nausea, vomiting, sedation, dizziness, constipation, respiratory depression, delayed mobilization, and the risk of persistent postoperative opioid use [3,11]. In the present cohort, the dexmedetomidine-based protocol was associated with both lower MME consumption and fewer PCA demands, suggesting that the observed early analgesic benefit may have reduced patient reliance on systemic opioid rescue.

The opioid-sparing effect observed in this study is broadly consistent with the pharmacological profile of dexmedetomidine and with emerging evidence from knee arthroplasty populations. Dexmedetomidine is a highly selective α_2_-adrenergic agonist with sedative, sympatholytic, and analgesic properties [12]. α_2_-adrenergic agonists have been associated with reductions in postoperative opioid consumption and pain intensity in perioperative settings [13]. Experimental and clinical studies also suggest that dexmedetomidine may prolong the effect of local anesthetics through peripheral and central mechanisms, including modulation of nociceptive transmission and prolongation of sensory blockade [14]. In arthroplasty populations, dexmedetomidine-containing periarticular infiltration has been reported to prolong early analgesic effects, reduce analgesic requirements, and improve recovery-related outcomes without a clear increase in adverse events [7,8]. However, extrapolation of these findings to HTO should be cautious because osteotomy procedures differ from arthroplasty in pain generators, rehabilitation priorities, and the importance of bone healing.

The present study may therefore provide procedure-specific preliminary evidence supporting dexmedetomidine-based opioid-free periarticular infiltration as a potential opioid-sparing strategy after medial opening-wedge HTO. Importantly, the protocol used in the present study avoided local morphine in the dexmedetomidine group while maintaining the same total injection volume and a comparable multimodal structure. This is clinically relevant because the continued inclusion of opioids in periarticular infiltration cocktails remains debated, particularly when the goal is to minimize perioperative opioid exposure. Nevertheless, the present findings should not be interpreted as proving that dexmedetomidine is definitively superior to morphine-containing infiltration. Because this was a retrospective, non-randomized comparison, the results indicate association rather than causation.

Safety is another important consideration when dexmedetomidine is used as an analgesic adjunct. Systemic dexmedetomidine may be associated with bradycardia, hypotension, and sedation, which are particularly relevant in perioperative patients [12]. In the present cohort, no statistically significant between-group differences were observed in nausea/vomiting, hypotension, bradycardia, allergic reactions, or other recorded analgesia-related adverse events. One case of bradycardia occurred in the dexmedetomidine group, but no significant safety signal was detected. However, the sample size was limited, and the study was not powered to evaluate uncommon adverse events. Therefore, the absence of a statistically significant safety difference should not be interpreted as definitive evidence of safety. Larger prospective studies with standardized hemodynamic and sedation monitoring are needed to better characterize the safety profile of dexmedetomidine-based periarticular infiltration after HTO.

A strength of the present study is that all HTO procedures were performed by the same senior surgeon using a standardized medial opening-wedge technique, the same implant system, similar fixation principles, and the same postoperative rehabilitation pathway. In addition, spinal anesthesia, postoperative PCA use, PCA settings, and follow-up intervals were standardized as much as possible. These features reduce variability related to surgeon technique, implant choice, rehabilitation protocol, and routine postoperative care. Furthermore, longitudinal pain trajectories were analyzed using linear mixed-effects modeling, which is appropriate for repeated postoperative pain measurements. The adjusted models also showed that the group × time interaction and the differences in opioid-related outcomes persisted after adjustment for available measured covariates, including age, BMI, preoperative VAS score, operative time, and tourniquet time. However, these adjusted analyses should be interpreted as adjustment for available covariates only, not as comprehensive control of all clinically relevant confounders.

Several limitations must be acknowledged. First, the retrospective and non-randomized design precludes causal inference. Treatment allocation was based on routine clinical practice rather than randomization, and residual selection bias or confounding by indication cannot be excluded. Second, several potentially important confounding variables were not consistently or reliably documented in the retrospective records and therefore could not be included in the adjusted models. These included ASA physical status, detailed comorbidity profiles, diabetes mellitus, hypertension, cardiovascular disease, psychiatric comorbidity, chronic opioid use, preoperative analgesic consumption, detailed sedation exposure, osteotomy correction angle, exact opening gap, planned mechanical axis correction, bone grafting details, and minor concomitant arthroscopic procedures. In addition, because treatment-group assignment could not be reliably determined and clinical information was incomplete for some excluded patients, formal comparison between the included and excluded cohorts was not possible. Third, the morphine and dexmedetomidine dose ranges used in the institutional protocols were relatively broad, and exact dose distributions were not consistently available for dose–response analysis. This may have introduced within-group heterogeneity. Fourth, rescue analgesic administration was based on routine clinical judgment rather than a strictly protocolized VAS threshold, which may have introduced provider-related variability. Fifth, the study focused on early postoperative analgesic outcomes and did not evaluate validated functional outcomes, patient satisfaction, sleep quality, mobilization milestones, radiographic union, osteotomy healing, cost-effectiveness, or long-term clinical recovery. Finally, although no significant safety signal was observed, the sample size was insufficient to assess rare adverse events or to establish definitive safety conclusions. Future prospective randomized studies should use standardized infiltration doses, predefined rescue analgesic criteria, systematic sedation and hemodynamic monitoring, and comprehensive collection of perioperative confounders. Such studies should also evaluate patient-centered recovery measures, functional outcomes, rehabilitation milestones, opioid-related adverse effects, radiographic osteotomy healing, and long-term clinical outcomes. These data are needed to determine whether the early opioid-sparing association observed with dexmedetomidine-based periarticular infiltration translates into meaningful functional benefits after HTO.

In summary, dexmedetomidine-based opioid-free periarticular infiltration was associated with modestly lower early postoperative pain scores, reduced 24 h systemic opioid consumption, and fewer PCA demands compared with a modified opioid-containing Ranawat cocktail after medial opening-wedge HTO. Because of the retrospective non-randomized design and the limited availability of several important confounding variables, these findings should be considered hypothesis-generating and should be confirmed in adequately powered prospective randomized trials.

## 5. Conclusions

In this retrospective comparative cohort study, dexmedetomidine-based opioid-free periarticular infiltration was associated with lower early postoperative VAS scores, reduced 24 h systemic opioid consumption, and fewer PCA demands compared with a modified opioid-containing Ranawat cocktail after medial opening-wedge high tibial osteotomy. However, the absolute VAS differences were modest, and pain trajectories converged by postoperative day 14. No significant safety signal was detected in this small cohort, although the study was not powered to evaluate rare adverse events. Because treatment allocation was not randomized and several potentially relevant confounders were not available for adjustment, these findings should be interpreted as associative and hypothesis-generating. Prospective randomized studies with standardized dosing protocols and comprehensive assessment of functional recovery, opioid-related adverse effects, and osteotomy healing are warranted.

## Figures and Tables

**Table 1:** Baseline and perioperative characteristics according to periarticular infiltration protocol.

Variable	Modified Ranawat (n = 40)	Dexmedetomidine (n = 40)	*p*-Value
Age (years), mean ± SD	53.29 ± 7.86	51.09 ± 7.85	0.213
Female sex, n (%)	16 (40.0)	18 (45.0)	0.821 †
BMI (kg/m^2^), mean ± SD	28.29 ± 3.20	27.77 ± 3.66	0.494
Left-sided surgery, n (%)	27 (67.5)	19 (47.5)	0.113 †
Operative duration (min), mean ± SD	78.58 ± 9.77	81.77 ± 10.40	0.161
Tourniquet time (min), mean ± SD	62.5 ± 8.7	64.2 ± 9.1	0.395
Preoperative VAS, mean ± SD	6.91 ± 0.82	6.97 ± 1.10	0.774

Values are presented as mean ± standard deviation unless otherwise indicated. BMI: body mass index; VAS: Visual Analog Scale. † Fisher’s exact test.

**Table 2:** Linear mixed-effects models evaluating postoperative VAS pain trajectories.

Effect	Unadjusted Model, F (Num df, Den df)	*p* Value	Adjusted Model, F (Num df, Den df)	*p* Value
Group	F(1, 76.3) = 4.79	0.032	F(1, 74.8) = 5.46	0.022
Time	F(5, 384.7) = 380.97	<0.001	F(5, 378.2) = 381.00	<0.001
Group × Time	F(5, 371.2) = 7.75	<0.001	F(5, 368.5) = 7.78	<0.001
Age	—	—	F(1, 74.8) = 0.01	0.933
BMI	—	—	F(1, 74.8) = 2.63	0.109
Preoperative VAS	—	—	F(1, 74.8) = 0.30	0.586
Operative time	—	—	F(1, 74.8) = 2.04	0.158
Tourniquet time	—	—	F(1, 74.8) = 0.09	0.760

Adjusted model included the available covariates of age, body mass index, preoperative VAS score, operative time, and tourniquet time. Degrees of freedom were estimated using the Satterthwaite approximation. BMI, body mass index; VAS, Visual Analog Scale.

**Table 3:** Postoperative analgesic outcomes and analgesia-related adverse events.

Outcome	Modified Ranawat Group (n = 40)	Dexmedetomidine Group (n = 40)	Mean Difference or Comparison	*p* Value
Total opioid consumption, MME	13.92 ± 1.79	9.78 ± 1.99	4.14 (95% CI, 3.30–4.98)	<0.001
PCA demand, times	9.63 ± 2.92	6.30 ± 3.16	3.33 (95% CI, 1.98–4.68)	<0.001
Rescue NSAID use, n (%)	8 (20.0)	10 (25.0)	—	0.790 †
Rescue tramadol use, n (%)	9 (22.5)	7 (17.5)	—	0.781 †
Nausea/vomiting, n (%)	2 (5.0)	3 (7.5)	—	1.000 †
Hypotension, n (%)	1 (2.5)	1 (2.5)	—	1.000 †
Bradycardia, n (%)	0 (0.0)	1 (2.5)	—	1.000 †
Allergic reaction, n (%)	2 (5.0)	1 (2.5)	—	1.000 †
Length of hospital stay, days	2.46 ± 0.33	2.29 ± 0.30	0.17 (95% CI, 0.03–0.31)	0.017

Values are presented as mean ± standard deviation or n (%). Mean differences are calculated as modified Ranawat group minus dexmedetomidine group. MME: morphine milligram equivalents; PCA: patient-controlled analgesia; NSAID: nonsteroidal anti-inflammatory drug; CI: confidence interval. † Fisher’s exact test.

## Data Availability

The data presented in this study are available from the corresponding author upon reasonable request. The data are not publicly available due to privacy and ethical restrictions.

## Supplementary Materials

The following supporting information can be downloaded at https://www.mdpi.com/article/10.3390/healthcare14162580/s1: Supplementary Table S1: RECORD checklist; Supplementary Table S2: Adjusted analysis of covariance models for postoperative opioid consumption and PCA demand. Ref. [9] is cited in the Supplementary Material.
