# Supplementary material for: Dexmedetomidine-Based Versus Opioid-Containing Periarticular Infiltration in High Tibial Osteotomy: A Retrospective Comparative Cohort Study
Source: Healthcare (Basel). 2026 Aug 17;14(16):2580. doi: 10.3390/healthcare14162580 (PMC13512448; doi:10.3390/healthcare14162580)
Supplement: Supplementary file 1 [file healthcare-14-02580-s001.zip › RECORD_Checklist_Supplementary_Table_S1.pdf]

Supplementary Table S1. RECORD Checklist

Checklist for the reporting of studies conducted using observational routinely collected health data.

Item No.	STROBE item	Location in manuscript	RECORD item	Location in manuscript
**Title and Abstract**				
1	Indicate the study design with a commonly used term in the title or abstract; provide an informative and balanced summary of what was done and found.	Title; Abstract	RECORD 1.1: Specify the type of data used and, when possible, the name of the database.	Abstract; Methods 2.1; Methods 2.7
			RECORD 1.2: Report the geographic region and timeframe within which the study took place.	Abstract; Methods 2.1
			RECORD 1.3: State whether linkage between databases was conducted.	Not applicable; no database linkage was performed
**Introduction**				
2	Explain the scientific background and rationale for the investigation being reported.	Introduction		
3	State specific objectives, including any prespecified hypotheses.	End of Introduction		
**Methods**				
4	Present key elements of study design early in the paper.	Methods 2.1		
5	Describe the setting, locations, and relevant dates, including periods of recruitment, exposure, follow-up, and data collection.	Methods 2.1; Methods 2.2		
6	Give eligibility criteria, sources and methods of participant selection, and methods of follow-up.	Methods 2.2; Figure 1	RECORD 6.1: Describe methods of study population selection, including codes or algorithms if used; if not possible, provide an explanation.	Methods 2.2; Figure 1
			RECORD 6.2: Reference validation studies of codes or algorithms used to select the population.	Not applicable; no administrative coding algorithm was used
			RECORD 6.3: If database linkage was performed, consider a flow diagram showing linkage and numbers at each stage.	Not applicable; no linkage across separate databases was performed
7	Clearly define all outcomes, exposures, predictors, potential confounders, and effect modifiers.	Methods 2.3-2.8	RECORD 7.1: Provide a complete list of codes or algorithms used to classify exposures, outcomes, confounders, and effect modifiers, or explain if unavailable.	Methods 2.2; Methods 2.7
8	For each variable of interest, give sources of data and details of assessment methods.	Methods 2.7		
9	Describe any efforts to address potential sources of bias.	Methods 2.4; Methods 2.8; Discussion		
10	Explain how the study size was arrived at.	Methods 2.2		
11	Explain how quantitative variables were handled in the analyses.	Methods 2.8		

Item No.	STROBE item	Location in manuscript	RECORD item	Location in manuscript
12	Describe all statistical methods, including those used to control for confounding.	Methods 2.8; Results 3.3; Results 3.5	RECORD 12.1: Describe the extent to which investigators had access to the database population used to create the study population.	Methods 2.2; Methods 2.7
12a	Describe methods used to control for confounding.	Methods 2.8; Results 3.5; Supplementary Table S2		
12b	Describe methods used to examine subgroups and interactions.	Methods 2.8; Results 3.3		
12c	Explain how missing data were addressed.	Methods 2.2; Results 3.1; Results 3.3		
12d	Explain how loss to follow-up was addressed.	Methods 2.2; Results 3.1; Figure 1		
12e	Describe sensitivity analyses, if performed.	Not applicable; no formal sensitivity analysis was performed		
			RECORD 12.2: Provide information on data cleaning methods used in the study.	Methods 2.7
			RECORD 12.3: State whether person-level, institutional-level, or other data linkage across two or more databases was included.	Not applicable; no database linkage was performed
**Results**				
13	Report the number of individuals at each stage of the study and give reasons for non-participation; consider use of a flow diagram.	Results 3.1; Figure 1	RECORD 13.1: Describe the selection of persons included in the study, including filtering based on data quality and data availability.	Results 3.1; Figure 1
14	Give characteristics of study participants and information on exposures and potential confounders.	Results 3.2; Table 1		
14b	Indicate the number of participants with missing data for each variable of interest.	Methods 2.2; Results 3.1; Results 3.3		
14c	Summarize follow-up time.	Methods 2.7; Results 3.3		
15	Report numbers of outcome events or summary measures over time.	Results 3.3; Results 3.4; Table 2; Table 3; Figure 2		
16	Give unadjusted and adjusted estimates with precision; clarify which confounders were adjusted for.	Results 3.3; Results 3.5; Table 2; Table 3; Supplementary Table S2		
17	Report other analyses, including subgroup, interaction, and sensitivity analyses.	Results 3.3; Results 3.5		
**Discussion**				
18	Summarize key results with reference to study objectives.	Discussion		
19	Discuss limitations, including potential sources of bias or imprecision.	Discussion; Limitations paragraph	RECORD 19.1: Discuss implications of using data not collected specifically for the research question, including misclassification bias, unmeasured confounding, missing data, and changing eligibility over time.	Discussion; Limitations paragraph

Item No.	STROBE item	Location in manuscript	RECORD item	Location in manuscript
20	Give a cautious overall interpretation of results considering objectives, limitations, multiplicity of analyses, similar studies, and relevant evidence.	Discussion; Conclusions		
21	Discuss the generalizability of the study results.	Discussion; Limitations paragraph		
**Other Information**				
22	Give the source of funding and the role of funders.	Funding		
			RECORD 22.1: Provide information on access to the study protocol, raw data, or programming code.	Data Availability Statement; Supplementary Materials

Abbreviations: BMI, body mass index; HTO, high tibial osteotomy; LMM, linear mixed-effects model; MME, morphine milligram equivalents; PCA, patient-controlled analgesia; RECORD, REporting of studies Conducted using Observational Routinely-collected health Data; STROBE, Strengthening the Reporting of Observational Studies in Epidemiology; VAS, Visual Analog Scale.

Reference: Benchimol, E. I., Smeeth, L., Guttmann, A., Harron, K., Moher, D., Petersen, I., Sorensen, H. T., von Elm, E., Langan, S. M., & the RECORD Working Committee. (2015). The REporting of studies Conducted using Observational Routinely-collected health Data (RECORD) statement. PLoS Medicine, 12(10), e1001885. https://doi.org/10.1371/journal.pmed.1001885
