# Supplementary material for: Dexmedetomidine-Based Versus Opioid-Containing Periarticular Infiltration in High Tibial Osteotomy: A Retrospective Comparative Cohort Study
Source: Healthcare (Basel). 2026 Aug 17;14(16):2580. doi: 10.3390/healthcare14162580 (PMC13512448; doi:10.3390/healthcare14162580)
Supplement: Supplementary file 1 [file healthcare-14-02580-s001.zip › Supplementary Table S2.pdf]

Supplementary Table S2

Supplementary Table S2. Adjusted analysis of covariance models for postoperative opioid consumption and PCA demand.

Outcome	Predictor / Covariate	F	p value	Partial η^2
**Total 24-hour opioid consumption, MME**	Treatment group	98.33	<0.001	0.574
	Age	0.002	0.963	0.000
	BMI	4.07	0.047	0.053
	Preoperative VAS score	0.003	0.957	0.000
	Operative time	1.92	0.170	0.026
	Tourniquet time	2.51	0.117	0.033
**PCA demand count**	Treatment group	21.54	<0.001	0.228
	Age	0.000	0.997	0.000
	BMI	2.44	0.123	0.032
	Preoperative VAS score	0.35	0.556	0.005
	Operative time	0.95	0.332	0.013
	Tourniquet time	0.55	0.462	0.007

Abbreviations: BMI, body mass index; MME, morphine milligram equivalents; PCA, patient-controlled analgesia; VAS, Visual Analog Scale.

Table note:
Analysis of covariance models were adjusted for age, body mass index, preoperative VAS score, operative time, and tourniquet time. Treatment group refers to the periarticular infiltration protocol: modified Ranawat cocktail versus dexmedetomidine-based cocktail. Because of the retrospective design, adjustment was limited to covariates that were consistently available in the medical records.
